# The index of rural access: an innovative integrated approach for measuring primary care access

**DOI:** 10.1186/1472-6963-9-124

**Published:** 2009-07-23

**Authors:** Matthew R McGrail, John S Humphreys

**Affiliations:** 1 Gippsland Medical School, Monash University, Northways Road, Churchill, Victoria, 3842, Australia; 2 School of Rural Health, Monash University, PO Box 666, Bendigo Central, Victoria, 3552, Australia,

## Abstract

**Background:**

The problem of access to health care is of growing concern for rural and remote populations. Many Australian rural health funding programs currently use simplistic rurality or remoteness classifications as proxy measures of access. This paper outlines the development of an alternative method for the measurement of access to primary care, based on combining the three key access elements of spatial accessibility (availability and proximity), population health needs and mobility.

**Methods:**

The recently developed two-step floating catchment area (2SFCA) method provides a basis for measuring primary care access in rural populations. In this paper, a number of improvements are added to the 2SFCA method in order to overcome limitations associated with its current restriction to a single catchment size and the omission of any distance decay function. Additionally, small-area measures for the two additional elements, health needs and mobility are developed. By utilising this improved 2SFCA method, the three access elements are integrated into a single measure of access. This index has been developed within the state of Victoria, Australia.

**Results:**

The resultant index, the Index of Rural Access, provides a more sensitive and appropriate measure of access compared to existing classifications which currently underpin policy measures designed to overcome problems of limited access to health services. The most powerful aspect of this new index is its ability to identify access differences within rural populations at a much finer geographical scale. This index highlights that many rural areas of Victoria have been incorrectly classified by existing measures as homogenous in regards to their access.

**Conclusion:**

The Index of Rural Access provides the first truly integrated index of access to primary care. This new index can be used to better target the distribution of limited government health care funding allocated to address problems of poor access to primary health care services in rural areas.

## Background

Although access to health care is recognised internationally as a fundamental human right [1,2], population access to health care services in many areas is inadequate [3,4]. Globally, many rural and remote communities, often characterized by high levels of need for health care because of their poor health status [5], face enormous access barriers (both spatial and aspatial) to health care services. Indeed, for many rural and remote populations, access to health care is the most important issue [6].

Within Australia, numerous programs have been introduced by governments aimed at reducing access difficulties confronting rural populations [7]. Most of these programs target the provision of primary care services, because these are the gateway to the health system for most health care seekers. Moreover, primary care services are most effective at reducing inequities within these populations [8,9]. To date, however, in the absence of a national index of access, funding for most of these Australian programs is based (rather inadequately) on one of two rural classifications as surrogate measures of access [10] – either the Rural Remote and Metropolitan Areas (RRMA) classification [11] which divides all Statistical Local Areas (SLAs) into three zones (metropolitan, rural and remote) and seven categories across these zones based on the size of the largest population centre within each SLA; or the Australian Standard Geographical Classification (ASGC) Remoteness index [12] which measures road distance (proximity) to five hierarchical levels of service centres (proxy for availability) based on population size alone, the aggregated scores of which are then separated into five hierarchical categories.

Both of these classifications categorise most of non-metropolitan Australia into a few large, apparently homogenous, areas. Moreover, key dimensions of access are not included within either of the RRMA or ASGC Remoteness classifications because this was never their intended application. Nor is either classification able to detect small area variations of access [13].

This article outlines the development of a new index of access to primary care services designed to provide a better and more sensitive measure of access to health care services, particularly in non-metropolitan areas. Importantly, this index integrates the key geographical aspects of distance and location that underpin existing rural classifications with critical aspatial barriers associated with accessing primary care services.

### Barriers to accessing primary care services

The first step to developing an integrated measure of access is to identify relevant spatial and aspatial barriers [14,15]. Access to health services is a function of several factors, including appropriate supply (availability), reasonable distance/time impedance to available services (proximity), the level and nature of need for those seeking care (health needs) and the ability of individuals to access care at a time of need (mobility) [16,17].

Inadequate availability or supply of rural health care services is the most obvious barrier to accessing services at times of need [14,18,19]. Since health services are not ubiquitously available [20], distance and/or time separation between population and health service locations represents an important access barrier for rural populations [21-24]. Regardless of geographical location, a population can only have good access if the availability of services is adequate. Similarly, a population can only have good access if services are located in reasonable proximity, regardless of availability. Thus availability and proximity must be considered together when measuring health service access, often referred to as "*spatial accessibility*" [25-27].

Since achieving equitable access is widely accepted as an important goal of health service planning [28-31], it follows that the *health needs *of populations also impact significantly on service availability. A population characterised by higher health needs requires relatively more services to maintain an equivalent level of access compared to a similarly sized population with lower health needs. Similarly, the *mobility *of a population is another important factor determining their ability to overcome the distance barrier. A key facilitator of mobility is an individual's access to transportation, which enables them to transcend distance when accessing services [32-34]. The addition of these two elements, health needs and mobility enables a broader spectrum of health care access to be captured rather than being simply a measure of geographical accessibility.

With the exception of Wang and Luo [35] and one Australian index which measures access to rural education services [36], there are no rural measures of health care access that integrate both spatial and aspatial barriers. The following Index of Rural Access is designed to take account of these barriers (service availability, proximity, health needs and mobility) which together differentiate access to primary care. Additional barriers [16], which may further explain utilisation differences, such as acceptability (individual chooses not to access services because of provider or organisation preference differences), attitudes (individual chooses not to access services because of health belief or attitude differences) and affordability (individual chooses not to access services because of out-of-pocket costs) are not incorporated in this index.

## Methods

**Figure 1:**
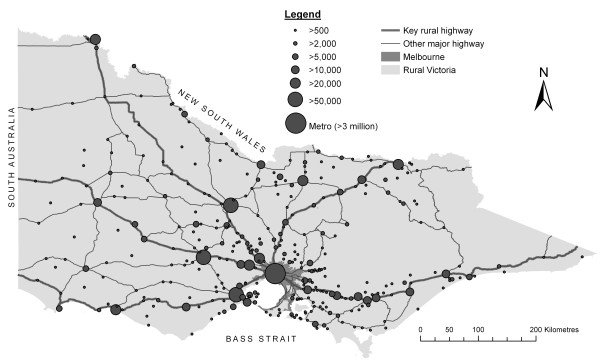
**Distribution of population centres within the state of Victoria**.

In constructing the Index of Rural Access, methods for measuring each individual barrier (spatial accessibility, health needs and mobility) are developed separately before integrating them as a single measure (index) of access. This methodology is defined over four stages which are outlined in detail below. Indicators used to measure each dimension were deliberately sourced from publicly available and regularly-updated datasets. Additionally, the need to measure small area variations of access in rural areas necessitates the use of the smallest feasible geographical unit for all indicators. This new index has been constructed for the state of Victoria, Australia (see Figure 1).

### Stage 1: Measuring spatial accessibility

Spatial accessibility measurement provides the platform upon which the overall measure of access is built. A recent advancement in the measurement of spatial accessibility for primary care in rural areas was the two-step floating catchment area (2SFCA) method [25,37]. Prior to this, spatial accessibility was commonly measured using population-to-provider ratios (for set regions), gravity models or nearest service [26,27]. Like population-to-provider ratios, the 2SFCA method does not utilise spatial movement data but rather it groups populations and health care services within a common boundary. Since spatial accessibility needs to capture proximity and availability together, two distinct elements are used in calculating the 2SFCA method – the *location *of both primary care services and the population, as well as the *number *of services and *size *of the population at each *location *[25,35]. What distinguishes the 2SFCA method is its use of floating catchment areas or 'windows' rather than set boundaries. In this way the 2SFCA method better concords with the assumption that the population will only use services within their catchment. Additionally, the 2SFCA maintains the representation of accessibility as easily-interpreted population-to-provider ratios. A more detailed description of calculating the 2SFCA method is described in Equations 1 – 2.

Population size and location data were obtained from the Australian Bureau of Statistics' (ABS) most recently available Census of Population and Housing of 2001 [38]. Until the very recent introduction of Mesh Blocks, Collection Districts (CD) were the smallest census-defined spatial units available in Australia used by the ABS as building blocks for collection, processing and output of data. In 2001, the average population size per CD was approximately 500 and an average areal size of 0.3 km^2 ^within Melbourne and 88 km^2 ^across rural Victoria. Because the focus of interest was on rural access, larger Statistical Local Areas (SLAs), with an average areal size of 30 km^2^, were used instead of CDs within metropolitan Melbourne, thereby significantly reducing the computations needed without losing data quality. All boundary files were available from the ABS and an approximate population point for each spatial unit was calculated using geometric centroids. Figure 1 shows the distribution or location of all population centres with more than 500 inhabitants within Victoria.

General Practitioners (GPs) were used to represent primary care services. All records for service providers listed as 'General Practice' within the state of Victoria were collected from the Medical Directory of Australia (MDA) [39], the most accurate and complete source of GP data. In addition, data from areas located within one hour travel from the Victorian border (that is, within South Australia and New South Wales) were also included to allow for demand from populations and services in other states. To ensure comprehensiveness, additional rural data were sourced from a number of other datasets and a random sample of the rural data was validated by the Rural Workforce Agency of Victoria. Self-reported GP workload from within the MDA was recorded as either full-time or part-time and up to three practice locations were recorded. Service locations were geocoded to a single latitude-longitude point, to enable their use within the Geographical Information System (GIS).

To complete the measurement of spatial accessibility, proximity between geocoded GPs and resident populations was calculated. Using ArcView 9.1, the 'Closest Facility' tool of the Network Analysis module was used to determine network routes and calculate proximity within a maximum catchment of 60 minutes. A file of all road sections of Victoria was obtained from Spatial Information Infrastructure [40] and travel time, rather than distance [41], was used as the measure of impedance by combining road section lengths and approximate travel speeds. All proximity data were imported into Microsoft Access 2003, where 2SFCA method calculations were performed, as briefly described below and in more detail by Wang and Luo [35]. Jenks natural break [42] method was used to define the classification levels for all choropleth maps.

The 2SFCA method provides a means of integrating these components in order to develop an accessibility measure. Firstly, Step 1 of the 2SFCA method computes a population-to-provider ratio, for each service location, by aggregating all population locations that are located within a defined threshold (catchment size).

**Step 1**:

(1)

*where S*_*j *_*is the number of full-time equivalent services at location j, P*_*i *_*is the number of residents at population location i and R*_*j *_*is the population-to-provider ratio for service j*

Then, Step 2 of the 2SFCA method also computes a population-to-provider ratio (access score), for each population location, by aggregating all service population-to provider ratios of services that are located within the same defined threshold (catchment size).

**Step 2**:

(2)

*where A*_*i *_*is the accessibility for population location i*

Despite the 2SFCA method providing an improved measure of spatial accessibility over other available methods [25,43], there remain two fundamental weaknesses. The first is its restriction to using only one catchment size for all areas and for both Steps 1 and 2, while the second weakness is the omission of any measure of the variation of proximity within a catchment itself. Our research addresses both these issues through developing four distinct improvements which enable the calculation of more realistic catchments and related impedance functions [44,45]. These improvements are summarised below, together with a brief justification shown in Table 1.

**Table 1:** Justifications for key decision points in the improvement of the 2SFCA method

**Decision point**	**Justification**
Initial catchment = 10 minutes (no decay)	In the Australian rural context, 10 minutes is viewed as an initial impedance that presents as no discernible barrier

Outer catchment limit = 60 minutes	The golden hour is a common rule of thumb, particularly in emergency care (which often is the primary care provider in rural Australia)

Distance decay occurs between 10 and 60 minutes	An impedance greater than 10 minutes is viewed as a significant and increasing barrier in the Australian rural context

Access is capped at the nearest 100 services	Populations are unlikely to access services beyond the nearest 100, thus capping provides a more realistic representation of the true catchment area

Step 1 (service) catchments are not always the same size	Services within large rural towns frequently do serve the populations of surrounding small rural towns. In contrast, small rural towns are unlikely to serve the populations of nearby large rural towns.

Different values for the initial catchment size (5 minutes, 15 minutes) and the capping level (50, 200) were also trialled. However, these were found to only make minimal difference to the overall access scores, the results of which are not shown in this paper.

a) Firstly, the addition of an impedance (distance-decay) function, f(d_ij_) within both service (Step 1) and population (Step 2) catchments, where d_ij _is the time between population *i *and service *j*, is required to more accurately reflect that services located at further distance/time barriers are less accessible to a population. Without this addition to the 2SFCA method, access is considered equal anywhere within the catchment which is clearly not the case in large rural catchments.

b) Secondly, the catchment should be split into two areas, so that distance-decay only begins after some initial period which offers no proximity barrier. For this study, the initial catchment used was 10 minutes (considered not to cause any significant impediment to accessing care) whilst the secondary catchment extended up to 60 minutes, within which distance-decay applied (Equation 3).

c) Thirdly, population access (Step 2) needs to be limited (capped) to the nearest 100 services (or 10 minutes for densely populated areas), beyond which the likelihood of access is deemed to be negligible, which closely follows Stouffer's concept of intervening opportunities [46]. The omission of this rule creates unstable results for fringe rural populations, due to them being dominated by nearby large metropolitan populations. The metropolitan populations have no need to access the rural fringe services because they have a large choice of nearby services to access within their metropolitan area.

d) Lastly, a few additional rules to the impedance function are required to more accurately reflect that service (Step 1) catchments are not always of equal size. In brief, large towns are likely to be providing services to smaller nearby populations whilst in contrast, small rural towns are not likely to be providing services to larger nearby populations [44,45]. Similar to improvement c), a smaller cap of 25 services is used in combination with the relative sizes of the service-providing town compared to the service-seeking town, in order for the impedance function at Step 1 to better 'model' the effect of nearby population size to the service catchment size.

Thus, the updated steps for the 2SFCA method are:

Impedance function, f(d_ij_), to implement improvements a) and b):

(3)

**Step 1**:

(4)

*where f*_*1*_*(d*_*ji *_*) takes the form of Equation 3, with the addition of a few rules as briefly described in improvement d)*

**Step 2**:

(5)

*where f*_*2*_*(d*_*ji *_*) takes the form of Equation 3*

### Stage 2: Measuring health needs

For the Index of Rural Access, health needs within each CD was approximated using sentinel predictor variables in combination with observed Disability Adjusted Life Years (DALYs). The accurate measurement of health needs is complex and difficult [47,48], and traditionally is a choice between a measure of observed health needs or predicted health needs. It follows that observed measures of actual illness or death events are commonly used, but these are often criticised as poor measures of a population's (future) health needs [49-52]. Instead, predictor variables of a population's health needs are often used [53-56], chiefly due to advantages including that the data are cheaper to access, more reliable and regularly updated, and importantly for this research, the data are available at a much smaller geographic scale, thereby enabling a sensitive small-area measure of health needs, particularly for rural populations.

In Victoria, Disability Adjusted Life Years (DALYs), which captures the contribution of both premature mortality and morbidity [57,58], have been calculated at the relatively small spatial unit of Local Government Areas (LGAs) [59]. It should be noted that with a median population of 35,000, LGAs are still significantly larger and more heterogeneous than CDs. For the Index of Rural Access, a regression model using DALYs as the outcome variable was used to determine a sentinel list of predictor variables. Most of the predictor variables were derived from the Advantage-Disadvantage (SEIFA) index which provides the best selection of indicators for predicting health needs [53,60], and closely matches variables used in other health needs measures [35,61,62]. Dimensions captured in SEIFA include family type and income, education, occupation and employment status, and household type. Six additional possible predictor variables were also identified, including percentage of the population who are Indigenous and percentage of the population speaking little or no English.

Before regression modelling was undertaken, the list of possible predictor variables was reduced from 37 to 16. This was achieved by eliminating one of predictor variable pairs with high collinearity (r>0.80) [63,64] as well as eliminating predictor variables not related to the outcome measure of health needs (r<0.30) [65,66]. Particularly with using small area (CD) counts, it was also necessary to standardise each variable by using a log transformation of standardised χ^2 ^values [61,67].

The following list shows the final set of significant predictor variables after using backward elimination in the regression model of DALY scores:

• % Persons aged 15 years or over having an advanced diploma or diploma qualification

• % Persons aged 15 years and over at university or other tertiary institution

• % Employed Males classified as 'Intermediate Production and Transport Workers'

• % Males (in labour force) unemployed

• % One parent families with dependent offspring only

• % Persons Indigenous

These six variables captured 72% (R^2^) of the variance in observed health needs using DALY scores, and become the sentinel indicators for our measure of health needs. One additional variable not included in the regression modeling process because DALY scores are already age-sex standardised, captures the age-sex population cohorts characterised by high health needs. Following the same methods as Wang and Luo [35] and Field [61], three groups identified as high need (0–4 male and female; 15–44 female; and 65+ male and female) were aggregated to create one additional population high health needs variable.

Principal Components Analysis was used to determine the most appropriate combination and weighting of the resultant seven correlated sentinel indicators of health needs [66,68]. Two components, with eigenvalues greater than one, explained approximately 60% of the variance. Not surprisingly, component one (41.4% variance explained), comprised of all six predictor variables significantly related to DALY scores, which were all indicators of socio-economic-status disadvantage. Component two (18.8% variance explained) was dominated by the high need age-sex indicator, with several other indicators loading moderately on this component. A single health needs indicator was calculated by combining the two components together using a 2:1 weighting, based on their relative eigenvalues.

### Stage 3: Measuring mobility

Mobility is an indicator of the population's ability to transcend distance. In western societies, approximately 80% of the population travel by car when accessing primary care [69-71], so it is reasonable to assume that the majority of the population with a mobility barrier are those without access to a vehicle. Using data available from the national census, the simplest indicator is the number of households that do not own a car.

Of the approximately 20% of the population who use other modes of transport, it is reasonable to assume that personal mobility (walking or riding) and public transport contribute approximately 10% each to the mobility barrier. In general, the young and the elderly are most restricted in their personal mobility when access is required. Following Field's study [61], a measure of low personal mobility is the population size aged either below 18 years or more than 75 years, data which are available from the national census. Public transport availability varies enormously between major metropolitan centres, urbanised towns and rural areas. The measurement of access to public transport has two dimensions, namely frequency of services and proximity to service points (e.g. train station). For this study, each population unit (CD) was rated 0–4 and 0–6 for frequency and proximity respectively and summed to give a public transport access rating of 0–10 using service information from Metlink & Viclink [72]. As expected, this indicator shows a strong rural-urban divide with urban areas relatively well served by public transport, whilst rural areas generally have significantly few options.

These three indicators (households without a car, individuals of low personal mobility and public transport availability) measure different aspects of mobility and correlations between them are small. However, each contributes to the overall mobility (disadvantage) which in mathematical terms, equates to a linear combination with weightings attached to each indicator. It follows that appropriate weightings are 80, 10 and 10, based on existing evidence showing that cars are the population's predominant transport mode when accessing primary care services. Finally, the three mobility indicators were standardised before combining by using a log transformation for the two census indicators and a simple rescaling of the public transport discrete rating.

### Stage 4: Integrating health needs and mobility within the 2SFCA method

Stage four involved combining the important health needs and mobility dimensions with the major spatial accessibility platform outlined above. The significance of health needs is its association with service demand. If a population has high health needs then the relative demand within that population is greater than indicated by only the raw population size. Currently by itself, the 2SFCA method takes no account of the spatial variation of health needs. However, as proposed by Yang, Goerge *et al*. [43], the effect of health needs can simply be integrated within the 2SFCA method by its direct relationship to the service population size.

The demand on a service as measured by Step 1 of the 2SFCA method was defined earlier in Equation 4. If the demand (population size) increases then the denominator will increase resulting in a decreased service-population ratio or R_j_. Thus, for areas of relatively high need, P_i _should be increased and for areas of relatively low need, P_i _should be decreased. Hence, Step 1 should be adjusted with the inclusion of a health needs measure as shown in Equation 6, where health needs of population *i *is less than one for areas of low need and health needs of population *i *is greater than one for areas of high need.

**Updated Step 1**:

(6)

Before health needs can be added to the 2SFCA method, its indicator requires a transformation to an appropriate scale. In Equation 6, it is seen that HN_i _is multiplied by P_i_, thus the midpoint of the transformed HN_i _scale should be one (equivalent to no change to demand). The key transformation decision is to select a cap for areas of extreme health needs (both high and low). For this study, extreme areas (CDs) of low health need or high health need were capped at approximately half or twice health needs respectively, because this approximates the variation of DALY scores upon which the health needs indicator was modelled, when compared at the LGA level [59]. Thus low health needs scores were transformed to a range of (0.5, 1.0) and high health needs scores were transformed to a range of (1.0, 2.0), maintaining the population-to-provider ratio format of R_j_.

The significance of mobility level is its effect on distance (proximity). If an area has a high mobility disadvantage then the population's ability to transcend the distance between themselves and available services is decreased. This effect of mobility can be integrated within the 2SFCA method by its direct relationship to the population's catchment size (Step 2). A similar recommendation was proposed by Yang, Goerge et al. [43] who claimed that the 2SFCA method could be improved by creating dynamic catchment areas depending on the population's level of car ownership. Hence, Step 2 should be adjusted with the inclusion of a mobility measure as shown in Equation 7, where Mob_i _is equal to one within the initial catchment (10 minutes), and is less than one in the secondary catchment for areas of low mobility.

**Updated Step 2**:

(7)

As with health needs, the mobility indicator needs to be transformed to an appropriate scale before its inclusion within the 2SFCA method. Lack of any mobility problem should be transformed to a value of one (equivalent to no change to access), whilst maximum mobility disadvantage should be transformed to a value significantly less than one which results in a decreased population catchment size. Limited empirical evidence suggests that mobility may be a significant barrier for up to 30% of the population [73], thus mobility was capped at 0.70. Within Equation 7, mobility disadvantage can potentially decrease a population's Step 2 catchment size by a maximum of 30%, though unsurprisingly only limited spatial variation of mobility was observed in Victoria [33].

## Results

**Figure 2:**
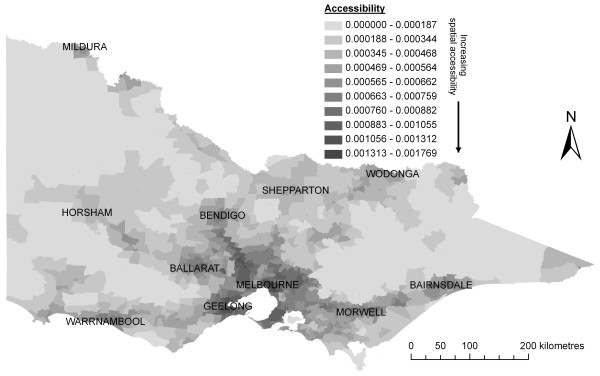
**Spatial accessibility of primary care in Victoria using the improved 2SFCA method**.

Figure 2 shows the results of applying the improved 2SFCA method (Equations 3–5) to measure spatial accessibility to primary care in Victoria. As expected, comparing this map against Figure 1 confirms that spatial accessibility is low in sparsely populated areas and generally higher in more populated areas where health services are more likely to be located.

**Figure 3:**
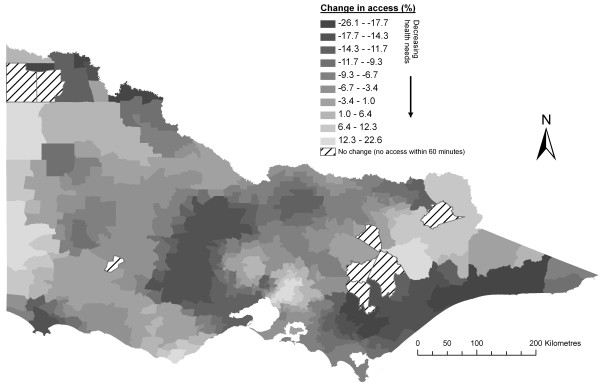
**Change to access scores after the addition of health needs**.

Figure 3 shows the change to spatial accessibility scores seen in Figure 2 after the integration of the health needs indicator. Areas showing the largest decrease in access correspond closely with areas of poorer health and socio-economic status and higher health needs [53,59].

**Figure 4:**
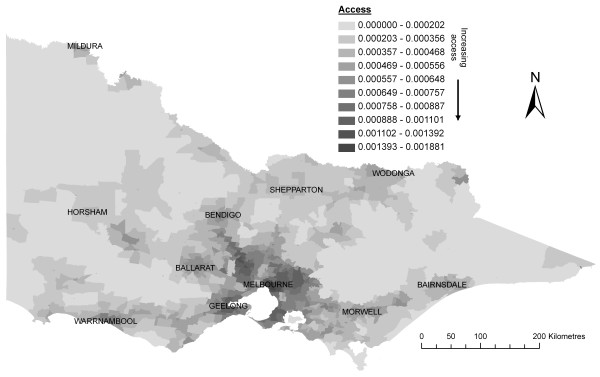
**The Index of Rural Access to primary care in Victoria**.

The final Index of Rural Access to primary care in Victoria is shown in Figure 4. This map has been calculated using a modified version of the 2SFCA method (Equations 3–7) which included the addition of four distinct improvements to the measure of spatial accessibility (a – d) as well as the integration of measures of health needs and mobility.

A demonstration of the greater precision of the Index of Rural Access in discerning variations of access to primary care services is shown in Table 2 by measuring its concordance with the currently used RRMA and ASGC Remoteness classifications for rural populations of Victoria.

**Table 2:** Concordance of the Index of Rural Access scores against two current classifications

		**Index of Rural Access** **(access scores in the form of provider-to-patient ratios)**					
		**>1:1200**	**1:1200–1:1600**	**1:1600–1:2000**	**1:2000–1:2500**	**1:2500–1:3000**	**<1:3000**
		**1**	**2**	**3**	**4**	**5**	**6**
**RRMA classification**	**Large Rural** **(25 k–99 k)**	0%	34%	44%	21%	1%	0%
	**Small Rural** **(10 k–25 k)**	0%	33%	30%	34%	2%	0%
	**Other Rural** **(<10 k)**	4%	16%	18%	17%	17%	29%

**ASGC Remoteness index**	**Inner Regional**	8%	34%	29%	14%	8%	8%
	**Outer Regional**	0%	5%	12%	36%	12%	36%

Table 2 shows that access to primary care is far from homogenous within each of the broad levels of the RRMA and ASGC Remoteness classifications, when compared against the Index of Rural Access. For the RRMA classification, the greatest spread of access scores is seen in 'Other Rural' areas – that is, those regions characterised by smaller dispersed populations. Whereas the RRMA classification treats these areas as being equal, the Index of Rural Access calculates a relatively uniform distribution of access scores within 'Other Rural' areas ranging between 1:1200 (equivalent to 0.000833) and much less than 1:3000 (0.000333). In 'Small Rural' and 'Large Rural' areas, though the range of access scores is less, there is still a uniform distribution of access scores ranging between 1:1200 and 1:2500 which once again shows the intra-regional heterogeneity of access to primary care in these areas. For the 'Inner Regional' area of the ASGC Remoteness classification, there is also a significant spread of access scores from the highest access right through to the lowest access.

## Discussion

Currently, most health incentive funding programs in Australia aimed at reducing access problems faced by rural and remote populations are based on either the RRMA or ASGC Remoteness classifications. The results shown in Table 2 highlight a major deficiency from using a purely geographical classification as the basis for resource allocation to address access problems. Existing geographical schemes classify large areas as homogenous and thus warranting equal treatment, whereas the use of a more appropriate and sensitive measure of access, such as the Index of Rural Access, indicates a very different scenario. The limitations associated with ongoing use of the RRMA and ASGC classifications have been recognised by both Liberal (conservative) and Labor governments responsible for funding of access initiatives [10,74]. However, until now, the complexity of developing a suitable index of access has apparently precluded the development of an acceptable solution.

The most powerful aspect of our new Index of Rural Access is its ability to identify areas with access disadvantage at a much finer geographical scale than previously existed. This advantage has been achieved through a number of key methodology design elements. Firstly, the incorporation of actual health service data enables a more accurate measure of availability and proximity as well as temporal changes to be easily captured. Secondly, an improved method to measure rural access was developed by introducing four improvements (a-d) to the 2SFCA method, which overcome its fundamental weaknesses of both failing to differentiate proximity within a catchment itself, and using a single catchment size for all population areas. Additionally, the important aspatial dimensions of access, health needs and mobility, have been integrated within the 2SFCA method. Lastly, all data have been collected and measured using the smallest available geographical unit (CD), which in combination with the first three design elements enables the Index of Rural Access to measure access at a much finer scale than alternative measures. As a result, application of this new index is likely to achieve a more equitable distribution of health service incentive funding within rural areas.

Although the Index of Rural Access has been constructed specifically for primary care services, its application is not restricted to measuring access to GPs. It is an appropriate tool (method) for measuring access for any primary health care service where populations are presented with a choice of services with overlapping service areas, such as dentists, pharmacists, optometrists and other allied health services.

The development of the Index of Rural Access could be further improved if stronger empirical evidence was available to guide some of the key decisions that are required in the development of any index such as this one. Alternative threshold values are available for consideration at several decision points, some of which were highlighted in Table 1, but currently there is little empirical evidence to guide definitive decisions. In the conduct of developing the Index of Rural Access, several alternatives were tested [45], the results of which showed that our choices were robust and realistic for rural and remote regions in geographically large countries such as Australia and Canada. Nonetheless, we recognise that applications of the Index of Rural Access in geographically small countries such as the United Kingdom and New Zealand or other more sparsely populated areas such as the state of Western Australia may warrant the choice of different threshold values. We invite further empirical research to help inform this decision-making.

We also acknowledge that the Index of Rural Access has not yet been externally validated as an improved measure of access in rural areas. Subsequent validation is very important, particularly prior to any comprehensive policy implementation changes based on this methodology. Such validation may come through feedback from the general population who experience access barriers first-hand, or perhaps through feedback from experts (such as rural workforce agencies) with a more intimate knowledge of service distribution. Furthermore, it is recognised that our approach to measuring health needs may not be ideal, but surrogate measures will always be required in situations where no adequate small area measures of observed health needs exists. We would also welcome validation through the application of this index in different settings, such as for different primary care service providers or in other locations both within and outside of Australia where better data may be available within government departments. Finally, we recommend that a useful validation would be to measure the association between the Index of Rural Access scores and age-sex standardised service utilisation rates, which in Australia is not currently possible because of limited access to such data at a sufficiently small spatial scale.

## Conclusion

Building on Luo and Wang's 2SFCA method, our Index of Rural Access provides the first truly integrated index of access to primary care services, in the Australian rural context. By combining the four key elements of availability of, and proximity to, services, health needs and mobility and through its calculation at the smallest feasible geographical scale using improved methods, the Index of Rural Access provides a more sensitive and appropriate measure of access, particularly for rural populations, than existing methods.

The potential application of the Index of Rural Access is very significant. In Australia, large amounts of government funding are allocated and distributed in the form of incentives designed to improve the recruitment and retention of the health workforce (especially GPs) in rural and remote areas where primary care workers are currently in short supply and difficult to recruit. Results from the Index of Rural Access in Victoria, have demonstrated the inadequacy of current classifications, upon which funding is based, to distinguish important geographical variation in access disadvantage. Thus, the Index of Rural Access provides a timely solution to the identification of areas of low or high access and can enable better targeted government funding.

## Competing interests

The authors declare that they have no competing interests.

## Authors' contributions

This study was conducted as part of a PhD thesis by MM. Both authors developed the idea for the study and MM conducted all aspects of the study under the close supervision of JH. JH aided the drafting of this manuscript. All authors read and approved the final manuscript.

## Pre-publication history

The pre-publication history for this paper can be accessed here:


